# Reviewer acknowledgement 2013

**DOI:** 10.1186/1749-799X-9-4

**Published:** 2014-02-06

**Authors:** Anish Raj Kadakia, Nicola Maffulli

**Affiliations:** 1 Northwestern University, Evanston, USA; 2 Queen Mary University of London, London, UK

## Abstract

**Contributing reviewers:**

The Editors of *Journal of Orthopaedic Surgery and Research* would like to thank all our reviewers who have contributed to the journal in Volume 7 (2013).

## 

Kashif Abbas

Pakistan

Mona Abbassy

Egypt

Joseph Abboud

United States of America

Atsuomi Aiba

Japan

Haruhiko Akiyama

Japan

Felix Angst

Switzerland

Mitsuhiro Aoki

Japan

Paul Arnold

United States of America

Henry Dushan Edward Atkinson

United Kingdom

George Babis

Greece

Nikolaos Bardakos

Greece

Mark Batt

United Kingdom

Christoph Becher

Germany

Rajni Bhan Safaya

India

Yu Binsheng

China

Jose Blanco

United Kingdom

Harald Böhm

Germany

Ferenc Boldizsar

Hungary

Peter Bonutti

United States of America

Lars Borris

Denmark

Stig Brorson

Denmark

Thomas Bucher

United Kingdom

Atilla Cıtlak

Turkey

Carina Cardemil

Sweden

Maurizio Catagni

Italy

Fabio Catani

Italy

Michel Chammas

France

Abhishek Chandra

United States of America

Shi-Min Chang

China

Christine Chappard

France

Jack Cheng

Hong Kong

Kin Wing Cheung

Hong Kong

Peter Ky Chiu

Hong Kong

Aurélien Courvoisier

France

Ross Crawford

Australia

William Cross

United States of America

Xu Cui

China

Carl D'arcy

Canada

Michael Daubs

United States of America

Ismail Demirkale

Turkey

Yunus Dogramaci

Turkey

Hans Dreyer

United States of America

Xavier Duralde

United States of America

Gabriella Dvorak

Austria

Amit Nandan Dhar Dwivedi

India

Turgay Efe

Germany

Chi Ho Jason Fan

Hong Kong

Fernando Fonseca

Portugal

Lonnie Froberg

Denmark

Qiang Fu

United States of America

Eisaku Fujimoto

Japan

Ashok Gavaskar

India

Julien Girard

France

Julie Glowacki

United States of America

Martin Gough

United Kingdom

Tarun Goyal

India

Stefan Greiner

Germany

Kirill Gromov

Denmark

Manuel Gutierres

Portugal

Andrew Hall

United Kingdom

Antoine Hamel

France

Helen Handoll

United Kingdom

Birgit Hanusch

United Kingdom

Langdon Hartsock

United States of America

Miroslav Haspl

Croatia

Heath Henninger

United States of America

Cila Herman

United States of America

Joachim Hillmeier

Germany

Florian Högel

Germany

Zhou Houxue

China

Kousuke Iba

Japan

Yasuo Ito

Japan

Vinay Jasani

United Kingdom

Nicolas Jaumard

United States of America

Tunku Kamarul

Malaysia

Nobuhiro Kamiya

United States of America

Heidi Kapstad

Norway

Yuichi Kasai

Japan

Orestis Katsamenis

United Kingdom

Michal Kedzierski

Poland

Daniel Kendoff

Germany

Dae Jin Kim

South Korea

Jin-Sung Kim

South Korea

Peter Kloen

The Netherlands

Jih-Yang Ko

Taiwan

Sanghun Ko

South Korea

Hirofumi Kosaka

American Samoa

David Kovacevic

United States of America

Matija Krkovic

United Kingdom

Sebastian Kuhn

Germany

Roel Kuijer

The Netherlands

Peter Kujath

Germany

Malhar Kumar

India

Paul Kuzyk

Canada

Kuo-An Lai

Taiwan

Jerzy Lasek

Poland

Binnaz Leblebicioglu

United States of America

Chianher Lee

Taiwan

Michael Lee

United States of America

Andre Leumann

Switzerland

Howard Levy

United States of America

Jinn Lin

Taiwan

Ruey Mo Lin

Taiwan

Guangwang Liu

China

Hwa-Chang Liu

Taiwan

Jun Liu

China

Olle Ljungqvist

Sweden

Dean Lorich

United States of America

Hsiao-Li Ma

Taiwan

Richard Ma

United States of America

Nicholas Maiden

Australia

Martin Majewski

Switzerland

Foued Makhlouf

France

Massimo Mariconda

Italy

Jonathan McCanless

United States of America

Meghan McGee-Lawrence

United States of America

Vishal Mehta

United States of America

Joao Batista Miranda

Brazil

Ralph Mobbs

Australia

Hitesh Modi

India

Michael A. Mont

United States of America

Hany Mourkus

United Kingdom

Christopher Mow

United States of America

Eric Nauman

United States of America

Vassilios Nikolaou

Greece

Andrej Nikoloski

Australia

Xiaohui Niu

China

Onder Ofluoglu

Turkey

Hyeong Seok Oh

South Korea

Francesco Oliva

Italy

Hiroshi Ozawa

Japan

Ali Fahir Ozer

Turkey

Johnny Padulo

Italy

Chun Hong Pang

Hong Kong

Stamatios Papadakis

Greece

George Paraskevas

Greece

Daniel Park

United States of America

Eui Kyun Park

South Korea

David Parker

Australia

Martyn Parker

United Kingdom

Stuart Phillips

Canada

Renata Prado

Brazil

Ravi Raj

India

Steven Rhemrev

The Netherlands

Yohan Robinson

Sweden

Cecilia Rogmark

Sweden

Hannes Rudiger

Switzerland

Fabio Santos

Brazil

Hanna Schell

Germany

Thomas Schildhauer

Germany

Florian Schmidutz

Germany

Werner Schmoelz

Austria

Lauren Schnabel

United States of America

Erik Schnaser

United States of America

Gundula Schulze-Tanzil

Germany

Rogier Simmermacher

The Netherlands

Paul Slosar

United States of America

Reuben Soh

Singapore

Nelson Soohoo

United States of America

Simon Steppacher

Switzerland

Karl Stoffel

Switzerland

Paul Stone

United States of America

Fabian Stuby

Germany

Tiansheng Sun

China

Robert Tashjian

United States of America

Guan Tay

Australia

Hidetomi Terai

Japan

Yoshinori Terashima

Japan

Gerhild Thalhammer

Austria

Ismail Remzi Tozun

Turkey

Cosimo Tudisco

Italy

Julio Urrutia

Chile

Christiaan Van Bergen

The Netherlands

Patrick Vavken

United States of America

Pascal-André Vendittoli

Canada

Juan Vivanco

United States of America

Diane Wagner

United States of America

Dirk Wähnert

Germany

Norimitsu Wakao

Japan

Bin Wang

China

Jeffrey Wang

United States of America

Qing Wang

China

Ashlee Watts

United States of America

Tim Weber

Germany

Rüdiger Weiss

Sweden

Jan Wieding

Germany

Britt Wildemann

Germany

Arne Wilharm

Germany

Philip Wolinsky

United States of America

Hajime Yamanaka

Japan

Lili Yang

China

Muneyoshi Yasuda

Japan

Takao Yasuhara

Japan

Hui Yu

China

Usama Zafar

Australia

Xu Zhang

China

Ying-Ze Zhang

China

Sun Zhenhui

China

Hong Zhou

Australia

Wierd Zijlstra

The Netherlands

Werner Zimmerli

Switzerland

David Zorman

Belgium

